# The effect of perioperative insulin treatment on cardiodepression in mild adiposity in mice

**DOI:** 10.1186/s12933-016-0453-y

**Published:** 2016-09-20

**Authors:** Chantal A. Boly, Etto C. Eringa, R. Arthur Bouwman, Rob F. P. van den Akker, Frances S. de Man, Ingrid Schalij, Stephan A. Loer, Christa Boer, Charissa E. van den Brom

**Affiliations:** 1 Department of Anesthesiology, Institute for Cardiovascular Research, VU University Medical Center, De Boelelaan 1117, 1081 HV Amsterdam, The Netherlands; 2 Department of Physiology, Institute for Cardiovascular Research, VU University Medical Center, Amsterdam, The Netherlands; 3 Department of Anesthesiology, Catharina Hospital, Eindhoven, The Netherlands; 4 Pulmonology, Institute for Cardiovascular Research, VU University Medical Center, Amsterdam, The Netherlands

**Keywords:** Insulin, Myocardial contraction, Myocardial perfusion, Adiposity, Perioperative period

## Abstract

**Background:**

While most studies focus on cardiovascular morbidity following anesthesia and surgery in excessive obesity, it is unknown whether these intraoperative cardiovascular alterations also occur in milder forms of adiposity without type 2 diabetes and if insulin is a possible treatment to improve intraoperative myocardial performance. In this experimental study we investigated whether mild adiposity without metabolic alterations is already associated with cardiometabolic dysfunction during anesthesia, mechanical ventilation and surgery and whether these myocardial alterations can be neutralized by intraoperative insulin treatment.

**Methods:**

Mice were fed a western (WD) or control diet (CD) for 4 weeks. After metabolic profiling, mice underwent general anesthesia, mechanical ventilation and surgery. Cardiac function was determined with echocardiography and left-ventricular pressure–volume analysis. Myocardial perfusion was determined with contrast-enhanced echocardiography. WD-fed mice were subsequently treated with insulin by hyperinsulinemic euglycemic clamping followed by the same measurements of cardiac function and perfusion.

**Results:**

Western-type diet feeding led to a 13 % increase in bodyweight, (p < 0.0001) and increased adipose tissue mass, without metabolic alterations. Despite this mild phenotype, WD-fed mice had decreased systolic and diastolic function (end-systolic elastance was 2.0 ± 0.5 versus 4.1 ± 2.4 mmHg/μL, p = 0.01 and diastolic beta was 0.07 ± 0.03 versus 0.04 ± 0.01 mmHg/μL, p = 0.02) compared to CD-fed mice. Ventriculo-arterial coupling and myocardial perfusion were decreased by 48 % (p = 0.003) and 43 % (p = 0.03) respectively. Insulin treatment in WD-fed mice improved echo-derived systolic function (fractional shortening 42 ± 5 % to 46 ± 3, p = 0.05), likely due to decreased afterload, but there was no effect on load-independent measures of systolic function or myocardial perfusion. However, there was a trend towards improved diastolic function after insulin treatment (43 % improvement, p = 0.05) in WD-fed mice.

**Conclusions:**

Mild adiposity without metabolic alterations already affected cardiac function and perfusion during anesthesia, mechanical ventilation and surgery in mice. Intraoperative insulin may be beneficial to reduce afterload and enhance intraoperative ventricular relaxation, but not to improve ventricular contractility or myocardial perfusion.

## Background

Adiposity is a characteristic of about 50 % of patients undergoing anesthesia and surgery [1], and a risk factor for the development of cardiovascular complications in the perioperative setting. On one hand this is due to the presence of systolic and diastolic dysfunction, in part caused by elevated cardiac output, increased left-ventricular (LV) wall stress and cardiac hypertrophy [2]. On the other hand, increased adiposity is associated with decreased coronary flow reserve, which is indicative of impaired myocardial perfusion [3]. Moreover, the metabolic and cardiovascular alterations in the overweight conditions may influence response to analgesia, anesthetics, mechanical ventilation and surgical stress [4, 5]. Clinical studies focusing on the impact of anesthesia, mechanical ventilation and surgery on myocardial function frequently focus on morbid obesity, while milder forms of increased adiposity are neglected as risk factor in the perioperative setting. However, even milder forms of obesity and the metabolic syndrome are associated with a higher incidence of postoperative morbidity, including cardiac events [6]. In obese, non-diabetic rats, adrenergic-mediated hemodynamic effects were distinctly affected by the volatile anesthetic isoflurane when compared to non-obese rats [7]. Moreover, diet-induced obesity in rats is associated with systolic dysfunction and myocardial perfusion disturbances when compared to lean animals, and sevoflurane anesthesia further aggravates this myocardial contractile response [8]. Since these animal studies suggest that even non-diabetic adiposity is associated with hemodynamic and cardiovascular disturbances in the perioperative period, more insight in the pathophysiology as well as possible treatment strategies are warranted.

In order to gain insight in the impact of mild adiposity on myocardial function during anesthesia and surgery, we investigated whether mild adiposity without metabolic alterations is already associated with intraoperative alterations in myocardial function in an experimental mouse model. We further hypothesized that mild adiposity is associated with cardiovascular alterations that can be restored by perioperative insulin treatment. Insulin has positive inotropic effects and it is known that high dose insulin improves cardiac function in animals with septic and hemorrhagic shock [9, 10] but also in patients undergoing cardiac surgery [11].

## Methods

### Animals

Animal experiments were approved by the Institutional Animal Care and Use Committee of the VU University (Amsterdam, the Netherlands. Permit number: ANES 12-04/ANES 13-04), and were conducted following the European Convention for the Protection of Vertebrate Animals used for Experimental and Other Scientific Purposes and the ARRIVE guidelines on animal research [12]. Five week old male C57BL/6J obtained from Charles River Laboratories mice were housed in a temperature-controlled room (20–23 °C; 40–60 % humidity) under a 12/12 h light/dark cycle starting at 6.00 am. Body weight was determined on a weekly basis. After 4 weeks of western-type diet feeding an oral glucose tolerance test was performed and mice subsequently underwent the in vivo protocol consisting of left ventricular (LV) pressure–volume measurements and (contrast-) echocardiography under anesthesia. At the end of the experiment, mice were euthanized by heart excision under anesthesia.

### Adiposity model and metabolic profiling

Mice were randomized into a group fed a western-type diet (D12079B, Research Diets New Brunswick, NJ) consisting of 17 % kcal protein, 43 % kcal carbohydrates and 41 % kcal fat, ad libitum for 4  weeks. The control group was fed a normal chow diet (Teklad 2016, Harlan, Horst, the Netherlands) with 16 % kcal protein, 4 % kcal fat and 80 % kcal carbohydrates for 4  weeks.

An oral glucose tolerance test (OGTT) was performed after 4 weeks of diet exposure. After overnight fasting, conscious mice received a bolus of glucose (2 g/kg) via oral gavage. Blood was drawn from a tail-cut immediately before glucose challenge and at 15, 30, 60, 90 and 120 min thereafter using a Precision Xceed Blood Glucose monitoring system (MediSense, UK). The area under the blood glucose curve (AUC) was used to compare glucose tolerance between western-type and control diet-fed mice.

In mice that had only undergone baseline measurements and no insulin treatment, blood was drawn from the right ventricle of the heart and plasma concentrations of fasting insulin, free fatty acids and triglycerides were measured using commercial ELISA and enzymatic measurement kits according to the manufacturer (LINCO research, St. Charles, MO; WAKO NEFA-C, Wako Pure Chemical Industries, Osaka, Japan).

### Anesthesia and mechanical ventilation

After 4 weeks of diet exposure, anesthesia was induced with fentanyl 0.5 mg/kg, midazolam 9.4 mg/kg and acepromazine 9.4 mg/kg injected intraperitoneally. Anesthesia was maintained by continuous intravenous infusion of fentanyl 0.1 mg/kg/h, midazolam 2.3 mg/kg/h and acepromazine 2.3 mg/kg/h via a 26G cannula in the tail vein. The trachea was intubated and the lungs were mechanically ventilated (maximal pressure 12–14 cm H_2_O; positive end-expiratory pressure, 2–4 cm H_2_O; respiratory rate 150 breaths/min) with oxygen-enriched air (40 % O_2_/60 % N_2_). Body temperature was maintained at 37 °C using a body-temperature controlled heating pad.

### Surgery and monitoring

A catheter was placed in the right jugular vein for infusion of contrast agent, insulin and/or glucose. The left carotid artery was cannulated for measurements of arterial blood pressure (Safedraw Transducer Blood Sampling Set, Argon Medical Devices, Texas, USA). Arterial blood pressure, ECG, heart rate and temperature were continuously recorded using PowerLab software (PowerLab 8/35, Chart 8.0; ADInstruments Pty, Ltd., Castle Hill, Australia).

### Experimental protocol

**Fig. 1:**
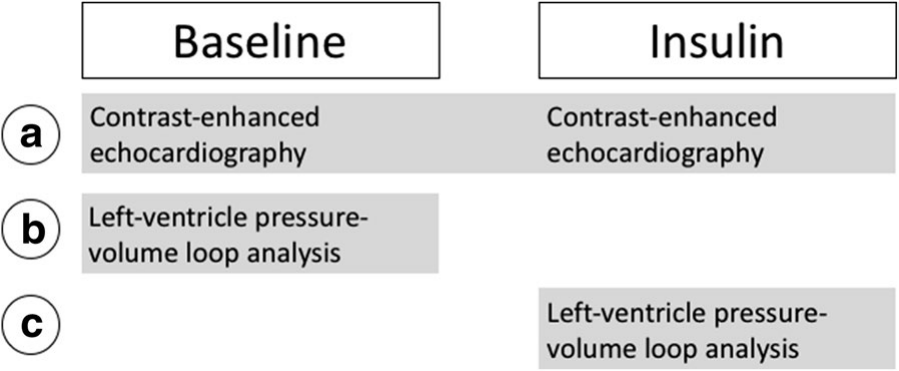
Experimental protocol. After induction of anesthesia and cannulation of vessels mice underwent contrast-enhanced echocardiography at baseline and after insulin treatment (group *A*), or pressure–volume loop analysis at baseline (group *B*) and after insulin treatment (group *C*)

After induction of anesthesia and cannulation of veins and arteries, mice underwent different experimental protocols, which are depicted in Fig. 1. (Contrast-enhanced) echocardiography measurements at baseline and during insulin treatment were paired (group A). However, pressure–volume loop measurements could not be paired due to the invasive nature of insertion of the Millar catheter in the LV (group B, C).

### (Contrast-enhanced) echocardiography

**Fig. 2:**
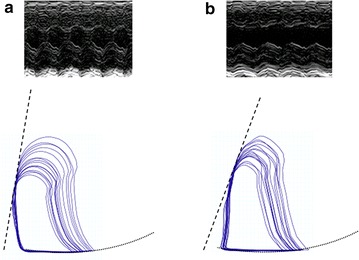
Typical examples of echocardiography and pressure–volume loops measurements in control (**a**) and western diet-fed (**b**) mice. Echocardiography windows are short axis midpapillary views which where used for estimation of fractional shortening, and demonstrated pressure volume loops were used for estimation of end-systolic (*striped line*) and end-diastolic (*dotted line*) pressure–volume relation estimations and related parameters

(Contrast-enhanced) echocardiography was performed using a Siemens (ACUSON, Sequoia 512) device equipped with a 14 MHz linear array transducer as described previously [8, 13]. Before infusion of contrast agent, contractile function was determined by visualizing the LV in short axis midpapillary view, and left ventricular end-systolic (ESD) and end-diastolic (EDD) diameters were determined in M-mode. LV systolic function is represented by fractional shortening (FS) which was calculated by the following equation: FS = (EDD − ESD)/EDD·100). All parameters were averaged over at least three cardiac contractile cycles. A typical example of echocardiography windows in control versus western diet-fed mice is shown in Fig. 2, upper part of panels a and b.

Contrast-echocardiography was performed as previously described [8, 13, 14]. Briefly, perfluorbutane filled microbubbles were continuously infused into the jugular vein and visualized in midpapillary short-axis views of the LV. After two minutes of microbubble infusion, perfusion recordings were taken with low acoustic power (mechanical index [MI] 0.19) for microbubble detection with a dynamic range of 50 dB, followed by a burst of high acoustic power (MI 1.8) for complete contrast destruction. Subsequently, on average 80 cardiac cycles of low MI images were acquired to allow contrast replenishment in the myocardium. All data were stored for offline analysis.

Custom-designed software was used for analysis of the estimate of myocardial perfusion (Matlab, 7.10, R2010A, MathWorks Inc. MA, USA) [14]. For each cardiac cycle, regions of interest were drawn in the end-systolic frame in the short axis view of the LV myocardium. Signal intensities from the frames after microbubble destruction were corrected for background noise by subtracting the signal intensity of the first frame after microbubble destruction (Y_0_). These intensities were then fitted [Y = Y_0_ + (*A*−Y_0_)·(1−exp^(−ß^·^x)^)] for calculation of microvascular blood volume *A* and the microvascular filling velocity ß, which corresponds to the capillary blood exchange rate. The estimate of myocardial perfusion was calculated as the product of *A* and ß.

### Pressure–volume loops

After induction of anesthesia a subcostal thoracotomy was performed and a pressure–volume Millar catheter (SPR 869, Millar Instruments, Houston, TX) was inserted in the LV via the apex. The Millar catheter continuously measures pressure and volume in the LV, and the volume signal was calibrated with stroke volume estimations that were obtained with echocardiography in the closed-chest situation. A ligature was placed under the inferior vena cava for transient preload reductions. Pressure–volume loops were measured in three series with vena cava occlusion-induced preload reduction. In a different group of mice, the same measurements were performed after hyperinsulinemic euglycemic clamping. Analysis of pressure volume loops was performed using PowerLab software (PowerLab 8/35, Chart 8.0; ADInstruments Pty, Ltd., Castle Hill, Australia). From steady state measurements, LV mean and end-systolic pressures as well as arterial elastance (E_a_), a measure for left ventricular afterload, were determined. From vena cava occlusion data, end-systolic elastance (E_es_) was determined as the slope of the end-systolic pressure–volume relationship, a load-independent measure for cardiac contractility. The slope of the end-diastolic pressure–volume relationship, Beta, was determined, which is considered a load-independent determinant of diastolic function. The ratio E_es_/E_a_ was determined as an estimate of ventriculo-arterial coupling, which describes adaptation of the LV in relation to the vasculature [15]. Typical examples of pressure–volume loops measurements during vena cava occlusion and related end-systolic and end-diastolic pressure–volume relations in control versus western-diet fed mice are shown in Fig. 2, the bottom of panels a and b.

### Insulin treatment

For insulin treatment with maintenance of euglycemic conditions, a hyperinsulinemic clamp was performed as described [16] after overnight fasting and under anesthesia. After determination of baseline glucose level a bolus of 100 mU/kg insulin was administered intravenously over 2 min followed by continuous infusion of insulin at a rate of 3 mU/kg/min for 60 min. Blood glucose levels were determined at intervals of 5 min and glucose infusion (20 % glucose diluted in NaCl) was adjusted to maintain euglycemia. After 60 min a steady state of euglycemia was achieved and mice were otherwise excluded from analysis.

### Data analysis

All data are presented as mean and standard deviation (SD). Statistical differences between the western-type diet and the control group were tested using a Student *t* test or two-way ANOVA for variables with multiple time points. The difference between insulin-stimulated conditions and the baseline condition in the obese group were tested with paired or Student t tests. A p < 0.05 was considered as statistically significant.

## Results

### Mild adiposity model

Characteristics of mice fed a control or western diet for 4 weeks are shown in Table 1. Western diet (WD) feeding led to an increase in caloric intake of almost 20 % compared to chow diet (p < 0.0001), which resulted in 13 % higher bodyweight (p < 0.0001) compared to controls and higher heart- and fatpad weights. There was no hyperglycemia, hyperinsulinemia or hyperlipidemia as determined under fasting conditions. Furthermore, an oral glucose tolerance test and hyperinsulinemic euglycemic clamp revealed the absence of glucose intolerance (area under the curve was 1823 ± 156 versus 1549 ± 68 in control mice, p = 0.13) and whole body insulin resistance (glucose infusion rate during hyperinsulinemic clamp was 24 ± 3 in WD-fed mice versus 25 ± 2 mg/min/kg in control mice, p = 0.73).

**Table 1:** Metabolic characteristics of control and WD-fed mice

	Control	WD-fed	p value
Body weight (g)	23.8 ± 0.8	27.0 ± 1.2	<0.0001*
Tibia length (mm)	17.2 ± 0.4	17.1 ± 0.4	0.66
Caloric intake (g/kg/week)	3.2 ± 0.1	3.8 ± 0.2	<0.0001*
Non-fasting blood glucose (mmol/L)	11.5 ± 1.9	11.8 ± 1.8	0.47
Fasting blood glucose (mmol/L)	6.2 ± 0.8	5.7 ± 0.6	0.18
Heart weight (mg)	103.1 ± 4.0	127.4 ± 16.0	0.01*
Left ventricular weight (mg)	79.5 ± 4.1	102.0 ± 15.8	0.001*
Right ventricular weight (mg)	19.1 ± 2.7	20.8 ± 4.0	0.34
Epididymal adipose tissue (mg)	184.3 ± 32.3	308.0 ± 119.1	0.01*
Perirenal adipose tissue (mg)	50.8 ± 10.2	84.5 ± 48.1	0.07
Epicardial adipose tissue (mg)	2.4 ± 1.0	7.2 ± 6.3	0.05
Plasma insulin (ng/mL)	2.71 ± 1.31	3.16 ± 1.06	0.85
Plasma triglycerides (ng/mL)	0.48 ± 0.05	0.55 ± 0.07	0.40
Plasma free fatty acids (nmol/mL)	0.09 ± 0.02	0.09 ± 0.02	0.51
Oral glucose tolerance test AUC	1549 ± 68	1823 ± 156	0.13
Hyperinsulinemic euglycemic clamp			
Steady state blood glucose (mmoll/L)	5.4 ± 0.2	5.6 ± 0.2	0.42
Glucose infusion rate (mg/min/kg)	25 ± 2.2	24 ± 2.8	0.73

Metabolic characteristics of control and WD-fed mice. *AUC* area under the curve. Data are presented as mean ± SD and n = 8–12 per group. Data were analyzed using a Student’s t test for between-group comparisons and 2-way ANOVA with repeated measurements for the oral glucose tolerance test (OGTT)

### Mild adiposity does not alter hemodynamics

Hemodynamics as measured under stable, anesthetized conditions are described in Table 2. Under anesthesia, there was no difference in mean arterial pressure between WD- and CD-fed mice (61 ± 19 versus 66 ± 17 mmHg in control mice, p = 0.34). Furthermore, although stroke volume showed a trend towards lower values in WD-fed mice (36 ± 2 versus 45 ± 4 μL in control mice p = 0.07), cardiac output was unaltered compared to controls.

**Table 2:** Baseline hemodynamics for control and WD-fed mice

	Control	WD-fed	p value
Mean arterial pressure (mmHg)	66 ± 17	61 ± 19	0.34
Heart rate (BPM)	604 ± 120	670 ± 65	0.18
Stroke volume (μL)	45 ± 11	36 ± 7	0.07
Cardiac output (mL/min)	26.8 ± 8.4	24.4 ± 3.6	0.46
End-systolic pressure (mmHg)	67 ± 16	61 ± 19	0.46

Data are presented as mean ± SD and n = 6–12 per group. Student t test, * p < 0.05

### Mild adiposity impairs cardiac function and perfusion

Cardiac function and perfusion parameters are described in Table 3. WD-feeding resulted in decreased systolic function as determined with echocardiography (fractional shortening 43 ± 6 % versus 56 ± 8 %, p ≤ 0.0001) as well as an almost 50 % decrease in pressure–volume loop-derived end-systolic elastance (E_es_ was 2.0 ± 0.5 versus 4.1 ± 2.4 mmHg/μL, p = 0.01) compared to CD-feeding. Afterload (E_a_) did not differ between groups. Ventriculo-arterial coupling (E_es_/E_a_) was diminished in WD-fed mice compared to controls (coupling ratio was 1.3 ± 0.6 versus 2.5 ± 1.4 in control mice, p = 0.003). Furthermore, WD-feeding increased diastolic Beta (0.07 ± 0.03 versus 0.04 ± 0.01 mmHg/μL, p = 0.02) compared to controls, which implies a stiffer ventricle. Myocardial perfusion was decreased by 43 % in WD-fed mice compared to CD-fed mice (p = 0.03).

**Table 3:** Myocardial function and perfusion parameters in WD-fed mice compared to controls

	Control	WD-fed	p value
Fractional shortening (%)	56 ± 8	43 ± 6	<0.0001*
End-systolic elastance (E_es_; mmHg/μL)	4.1 ± 2.4	2.0 ± 0.5	0.01*
Arterial elastance (E_a_; afterload; mmHg/μL)	1.6 ± 0.4	1.8 ± 0.9	0.51
Ventriculo-arterial coupling (E_es_/E_a_ ratio)	2.5 ± 1.4	1.3 ± 0.6	0.003*
Beta (diastolic stiffness; mmHg/μL)	0.04 ± 0.01	0.07 ± 0.03*	0.02*
Myocardial perfusion			
Microvascular flow velocity β (sec^−1^)	0.49 ± 0.20	0.59 ± 0.30	0.24
Microvascular blood volume A (A.U.)	0.014 ± 0.006	0.008 ± 0.005*	0.006*
Estimate of myocardial perfusion (A × β)	0.007 ± 0.005	0.004 ± 0.003*	0.03*

Data are presented as mean ± SD and n = 6–12 per group. Student t test, * p < 0.05

### Insulin treatment improves diastolic function in mildly adipose mice

**Fig. 3:**
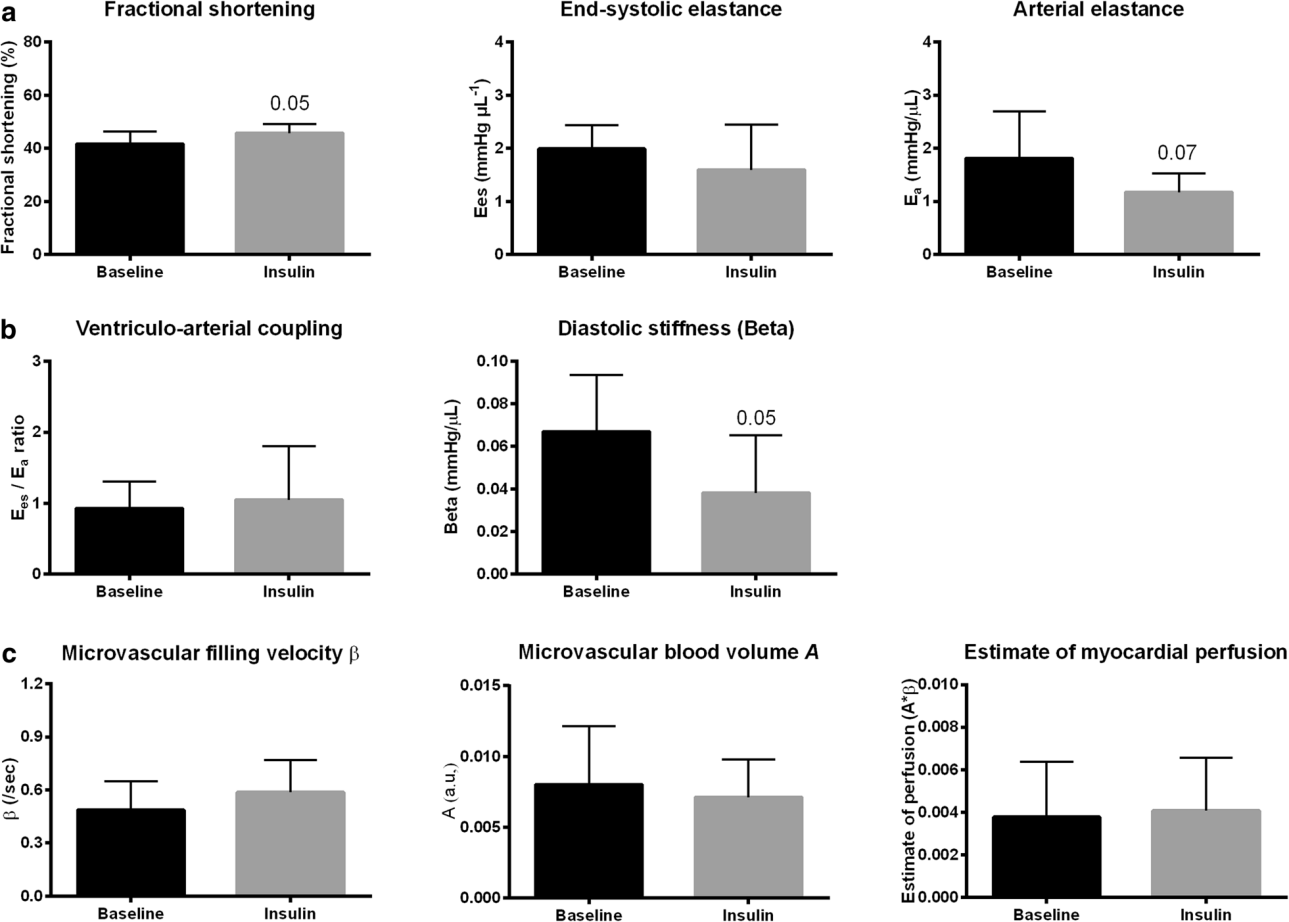
Cardiac function and perfusion response to insulin treatment in WD-fed mice. Cardiac systolic (**a**), and diastolic (**b**) function and perfusion (**c**) responses to insulin treatment. Data are mean ± SD and n = 7–10 per group. Paired t test for perfusion-related parameters, Student t test for other parameters, *p < 0.05

Hemodynamics and cardiac responses to insulin treatment in WD-fed mice are described in Table 4 and visualized in Fig. 3. After 1 h of insulin treatment, euglycemia was maintained at 5.6 ± 0.17 mmol/L. Mean arterial blood pressure and cardiac output remained unaltered during insulin treatment, despite a decrease in heart rate. However, insulin decreased end-systolic pressure (61 ± 19 mmHg to 43 ± 8 mmHg, p = 0.03), and there was a trend towards lower afterload (E_a_ decreased from 1.8 ± 0.9 to 1.2 ± 0.4 mmHg/μL, p = 0.07) during insulin treatment. While systolic echocardiographic parameters improved slightly in WD-fed mice during insulin treatment (fractional shortening increased from 42 ± 5 % to 46 ± 3, p = 0.05), there was no improvement in end-systolic elastance (E_es_ was 1.6 ± 0.3 versus 2.0 ± 0.1 mmHg/μL at baseline) nor in ventriculo-arterial coupling. Furthermore, there was a trend towards decreased Beta during insulin treatment (−43 % from 0.07 ± 0.01 to 0.04 ± 0.01 mmHg/μL p = 0.05), indicating improved diastolic function. Finally, myocardial perfusion was unaffected by insulin treatment in WD-fed mice.

**Table 4:** Hemodynamic response during insulin treatment in WD-fed animals

	Baseline	After insulin treatment	p value
Mean arterial pressure (mmHg)	61 ± 19	63 ± 12	0.91
Heart rate (BPM)	669 ± 65	593 ± 73	0.045*
Stroke volume (μL)	36 ± 7	39 ± 10	0.45
Cardiac output (mL/min)	24.4 ± 3.6	23.1 ± 6.4	0.58
End-systolic pressure (mmHg)	61 ± 19	43 ± 18	0.03*

Data are presented as mean ± SD and n = 7–10 per group. Student t test, * p < 0.05

## Discussion

In this study we demonstrated that a mild, early stage of diet-induced adiposity is already associated with decreased systolic and diastolic function, diminished ventriculo-arterial coupling and impaired myocardial perfusion during anesthesia, mechanical ventilation and surgery in mice. In this phenotype, intraoperative insulin treatment improved echo-derived systolic function, decreased afterload and showed a trend towards improved diastolic function. However, there was no effect on load-independent measurements of systolic function or myocardial perfusion. These combined findings suggest mild adiposity already compromises cardiac function during anesthetized and mechanically ventilated conditions, and that intraoperative insulin treatment may be applied to positively modulate loading conditions and ventricular relaxation, but not to influence cardiac contractility.

### Mild obesity is associated with intraoperative myocardial depression

In the present study we timed diet exposure to induce an early stage of adiposity without insulin resistance, so that the effect of early overweight conditions without confounding metabolic alterations could be studied. We demonstrated that intraoperative myocardial depression is already present in this early stage. Therefore, mildly adipose patients may be at increased risk for perioperative cardiac alterations, even when presenting in the early stage without metabolic alterations.

In literature, conflicting results have been reported regarding the effect of diet-induced adiposity on systolic function. Some report decreased [8, 13, 17, 18] others unchanged [19] or even improved [20] systolic function. Studies on cardiac function in obesity in the perioperative setting are rare, but have shown additional cardiodepressive effects of sevoflurane in diet-induced obese rats [8]. In our mild phenotype we found impaired fractional shortening but also load-independent end-systolic elastance, indicating that this is a true contractility effect. Decreased ventriculo-arterial coupling furthermore indicates inadequate adaptation of the heart to the vasculature. Importantly, exposure to general anesthesia and mechanical ventilation in combination with surgical stress in our study, may have exacerbated subclinical systolic dysfunction that would otherwise remain undetected.

Diastolic dysfunction is a consistent finding in diet-induced adiposity, and is the predominant cardiac feature in earlier stages of human obesity [2]. The increased slope of the end-diastolic pressure–volume relation in our study indicates increased ventricular stiffness, which is a finding consistent with several other animal studies on diet-induced adiposity [17, 18], although to our knowledge this has not been described in this early stage before.

Cardiac function relies on adequate myocardial perfusion [21], for which microvascular function and responsiveness are important determinants. In this study we demonstrated impaired myocardial perfusion in mild adiposity, indicating that myocardial vascular dysfunction is an early phenomenon in diet-induced adiposity. Impaired vasodilatory responses have earlier been demonstrated in isolated hearts of obese mice fed a high fat diet for 8 weeks [22]. Moreover, rats exposed to high fat diet for 6 weeks had impaired microvascular responsiveness to adenosine administration [19]. In overweight, obese [3, 23] and morbidly obese [23, 24] subjects, endothelium-dependent myocardial perfusion responses to cold pressure test as well as the response to dipyridamole were blunted. These findings indicate impaired microvascular responsiveness, and disturbances in microvascular function have been related to hyperglycemia, dyslipidemia and insulin resistance [25, 26]. Interestingly, in this study none of these were characteristics of our model, indicating that impairments of myocardial perfusion and function occur independently from metabolic defects in early stages of increased adiposity.

### Effect of intraoperative insulin treatment

The increasing prevalence of overweight and obese patients that undergo surgery under anesthesia and the myocardial depression demonstrated in the described mouse model warrant treatment strategies to optimize perioperative cardiac function. Insulin has been shown to improve cardiac function in the setting of acute myocardial infarction, ischemia and reperfusion injury, sepsis and hemorrhagic shock [9, 10, 27, 28]. In addition, perioperative insulin treatment was beneficial in cardiothoracic surgery [11, 29, 30] although another study showed no benefit in cardiothoracic surgery [31]. Up to now, no studies have investigated how insulin influences perioperative myocardial depression in overweight and obesity, but we hypothesized a beneficial effect. While most studies have used glucose-insulin-potassium (GIK) as insulin treatment, in this study we used hyperinsulinemic euglycemic clamping for this purpose because maintenance of euglycemia eliminates the possible effects of hypo- and hyperglycemia on myocardial perfusion and function itself [32]. Furthermore, insulin itself and not glucose or potassium was shown to be the factor responsible for the beneficial effects of GIK in a previous study [33].

In the present study, insulin treatment improved intraoperative fractional shortening, but not load-independent end-systolic elastance in mildly adipose mice. In combination with decreased afterload and end-systolic pressure during hyperinsulinemia, this effect is most likely due to peripheral vasodilation, which is in concordance with previous findings [29, 34, 35]. Most studies that demonstrated a benefit of insulin on perioperative cardiac function have used load-dependent parameters such as cardiac index [35–37], while others who studied load-independent measures such as myocardial strain, showed no effect [31]. This supports our findings that insulin mainly affects loading conditions, but not load-independent measurements of contractility. In this study, there was a trend towards improved diastolic function after insulin treatment, which is in agreement with earlier studies in lean animals [38]. We did however not observe an increase in myocardial perfusion upon insulin treatment, while insulin is known as a vasodilating agent in the myocardium [39]. Impaired vasodilation to insulin has however been demonstrated in other tissues in obesity, and may be consistent with the presence of microvascular dysfunction [16].

Taken together, these results suggest that intraoperative insulin treatment does not improve systolic function or myocardial perfusion, but decreases afterload and may improve ventricular relaxation when administered intraoperatively to mildly adipose mice with cardiodepression. While decreased systemic vascular resistance and afterload may reduce cardiac work load, it may also cause hypotension which could negatively affect tissue perfusion. However, we did not observe a significant effect on blood pressure and myocardial perfusion in this study. Although we observed no effects on systolic function, the trend towards a beneficial effect on diastolic function that we observed may on itself be relevant in the perioperative period. Diastolic dysfunction is an important risk factor in the perioperative period, as hemodynamic changes such as arrhythmias and tachycardia may affect diastolic filling, and existing diastolic dysfunction can acutely decompensate in the perioperative period [40].

## Conclusions

In conclusion, mild adiposity is already associated with intraoperative myocardial depression in mice. Intraoperative insulin treatment improved echo-derived systolic function, decreased afterload and showed a trend towards improved diastolic function, but not load-independent measurements of systolic function or myocardial perfusion. These combined findings suggest that the early stage of adiposity without metabolic alterations already compromises cardiac function during anesthetized and mechanically ventilated conditions, and that intraoperative insulin treatment may be applied to modulate loading conditions and ventricular relaxation, but not to influence cardiac contractility.
